# Advances and opportunities in unraveling cold‐tolerance mechanisms in the world's primary staple food crops

**DOI:** 10.1002/tpg2.20402

**Published:** 2023-11-13

**Authors:** Sofora Jan, Sachin Rustgi, Rutwik Barmukh, Asif B. Shikari, Brenton Leske, Amanuel Bekuma, Darshan Sharma, Wujun Ma, Upendra Kumar, Uttam Kumar, Abhishek Bohra, Rajeev K. Varshney, Reyazul Rouf Mir

**Affiliations:** ^1^ Division of Genetics & Plant Breeding Faculty of Agriculture (FoA) SKUAST‐Kashmir, Wadura Campus Sopore Kashmir India; ^2^ Department of Plant and Environmental Sciences Clemson University Florence South Carolina USA; ^3^ Center of Excellence in Genomics and Systems Biology International Crops Research Institute for the Semi‐Arid Tropics (ICRISAT) Hyderabad India; ^4^ Department of Primary Industries and Regional Development South Perth Western Australia Australia; ^5^ Centre for Crop & Food Innovation State Agricultural Biotechnology Centre Food Futures Institute Murdoch University Murdoch Western Australia Australia; ^6^ College of Agronomy Qingdao Agriculture University Qingdao China; ^7^ Department of Plant Science Mahatma Jyotiba Phule Rohilkhand University Bareilly Uttar Pradesh India; ^8^ Borlaug Institute for South Asia (BISA) Ludhiana Punjab India

## Abstract

Temperatures below or above optimal growth conditions are among the major stressors affecting productivity, end‐use quality, and distribution of key staple crops including rice (*Oryza sativa*), wheat (*Triticum aestivum*), and maize (*Zea mays* L.). Among temperature stresses, cold stress induces cellular changes that cause oxidative stress and slowdown metabolism, limit growth, and ultimately reduce crop productivity. Perception of cold stress by plant cells leads to the activation of cold‐responsive transcription factors and downstream genes, which ultimately impart cold tolerance. The response triggered in crops to cold stress includes gene expression/suppression, the accumulation of sugars upon chilling, and signaling molecules, among others. Much of the information on the effects of cold stress on perception, signal transduction, gene expression, and plant metabolism are available in the model plant *Arabidopsis* but somewhat lacking in major crops. Hence, a complete understanding of the molecular mechanisms by which staple crops respond to cold stress remain largely unknown. Here, we make an effort to elaborate on the molecular mechanisms employed in response to low‐temperature stress. We summarize the effects of cold stress on the growth and development of these crops, the mechanism of cold perception, and the role of various sensors and transducers in cold signaling. We discuss the progress in cold tolerance research at the genome, transcriptome, proteome, and metabolome levels and highlight how these findings provide opportunities for designing cold‐tolerant crops for the future.

## Core Ideas

- Cold stress significantly affects plant growth and development, particularly in staple food crops (rice, wheat, and maize) that are often grown in regions with cold climates.
- Understanding the physiological, biochemical, and molecular mechanisms that plants use to respond and adapt to cold stress is crucial for developing cold‐tolerant varieties of staple food crops.
- Advances in various technologies, such as transcriptomics, proteomics, and gene editing, have provided insights into the genetic and molecular mechanisms underlying cold tolerance in staple food crops.
- The knowledge gained from these technological advancements can be applied to develop new breeding strategies and crop improvement techniques to produce cold‐tolerant varieties of staple food crops ensuring food security in regions that experience cold stress.

**Abbreviations**
ABA: abscisic acid
BRs: brassinosteroids
bZIP: basic leucine zipper
CBF: C‐repeat/DRE‐binding factor
CDPKs: calcium‐dependent protein kinases
CKs: cytokinins
CMLs: calmodulin‐like proteins
COR: cold‐responsive
DBFs: dehydration responsive element‐binding factors
DHN: dehydrin
ETH: ethylene
GAs: gibberellins
GBS: genotyping‐by‐sequencing
GWAS: genome‐wide association study
ICE1 and ICE2: Inducer of CBF Expression
JA: jasmonic acid
LEA: late embryogenesis abundance
MAPK: mitogen‐activated protein kinase
MKKKs: MAPK kinase
MKKs: MAPK kinases
PM: plasma membrane
QTLs: quantitative trait loci
RBA: responsive to abscisic acid
ROS: reactive oxygen species
SA: salicylic acid
SNPs: single nucleotide polymorphisms

## 1 INTRODUCTION

Increasing climatic fluctuations threaten world food security as these are the primary drivers of abiotic and biotic stresses that limit agricultural production (Rosenzweig et al., 2014). Abiotic stresses, such as episodes of excessive cold or heat, precipitation or drought, and soil salinity or sodicity represent some of the most common types of stresses that plants experience in response to climate change (Ashraf et al., 2018; Barmukh et al., 2022; Soren et al., 2020; Varshney, Barmukh et al., 2021). Temperature fluctuations, particularly episodes of extreme cold, can lead to chill injury in major cereal crops such as wheat (*Triticum aestivum*), rice (*Oryza sativa*), and maize (*Zea mays* L.). These crops are either not naturally adapted to or have not been specifically bred for such cold conditions (Dolferus, 2014; Janksa et al., 2010; Solanke et al., 2008). At sub‐zero conditions, the formation of ice crystals, alterations in the permeability of biological membranes, and the generation of reactive oxygen species (ROS) take place, intracellularly or extracellularly. These changes result in a combination of symptoms like poor germination, reduced seedling vigor or stunted growth, reduced leaf size, leaf yellowing and withering, reduced tillering, poor root proliferation, disturbed plant water relations, impeded nutrient uptake, premature heading, increased seed abortion, and reduced seed size leading to reduced yield (Andaya &, Tai 2006; Hassan et al., 2021; Li et al., 2015; Oliver et al., 2002; Wang et al., 2013).

Being sessile organisms, plants must with stand temperature extremes in their habitats. The losses to cold stress can be minimized via a combination of breeding for cold tolerance and management practices (Rihan et al., 2017). Cold tolerance can be achieved by the expression of cold‐responsive genes; accumulation of hydrophilic proteins, soluble sugars, and osmolytes; metabolic readjustment; lipid membrane remodeling; and activation of ROS scavenging systems (Juurakko et al., 2021). There is abundant literature available showcasing the effects of cold stress on plant metabolism, perception and signal transduction, gene expression, and defense mechanisms (Chinnusamy et al., 2010; Kazemi et al., 2018; Sanghera et al., 2011; Solanke et al., 2008; Thakur & Nayyar, 2013; Yadav, 2010). However, these studies are mainly focused on the model plant *Arabidopsis thaliana*. Unfortunately, most economically important cereals are sensitive to low temperatures, which causes significant losses in crop yields, but there are very few studies that focus on how cereals perceive low‐temperature stress and acclimatize to such stressful climatic conditions. Therefore, there is a need to synthesize the information from model plants and utilize it in cereals to develop cold‐tolerant varieties.

In this review, we summarize some key research findings on how extreme climatic events, particularly cold stress, negatively affect the normal growth, development, and yield of the world's primary staple crops including rice, wheat, and maize. First, we describe how cold‐induced disruptions affect the morphophysiological and metabolic processes, which lower grain yield and grain quality in these crops. Next, we explain how these crops sense cold stress and respond to it by altering their genomes, transcriptomes, proteomes, lipidomes, and metabolomes, which enable them to endure cold stress without negatively affecting their growth and development. Finally, we highlight innovations in “omics” approaches for improving cold stress tolerance in staple crops.

## 2 EFFECT OF COLD STRESS ON MORPHOPHYSIOLOGICAL AND METABOLIC PROCESSES

**FIGURE 1:**
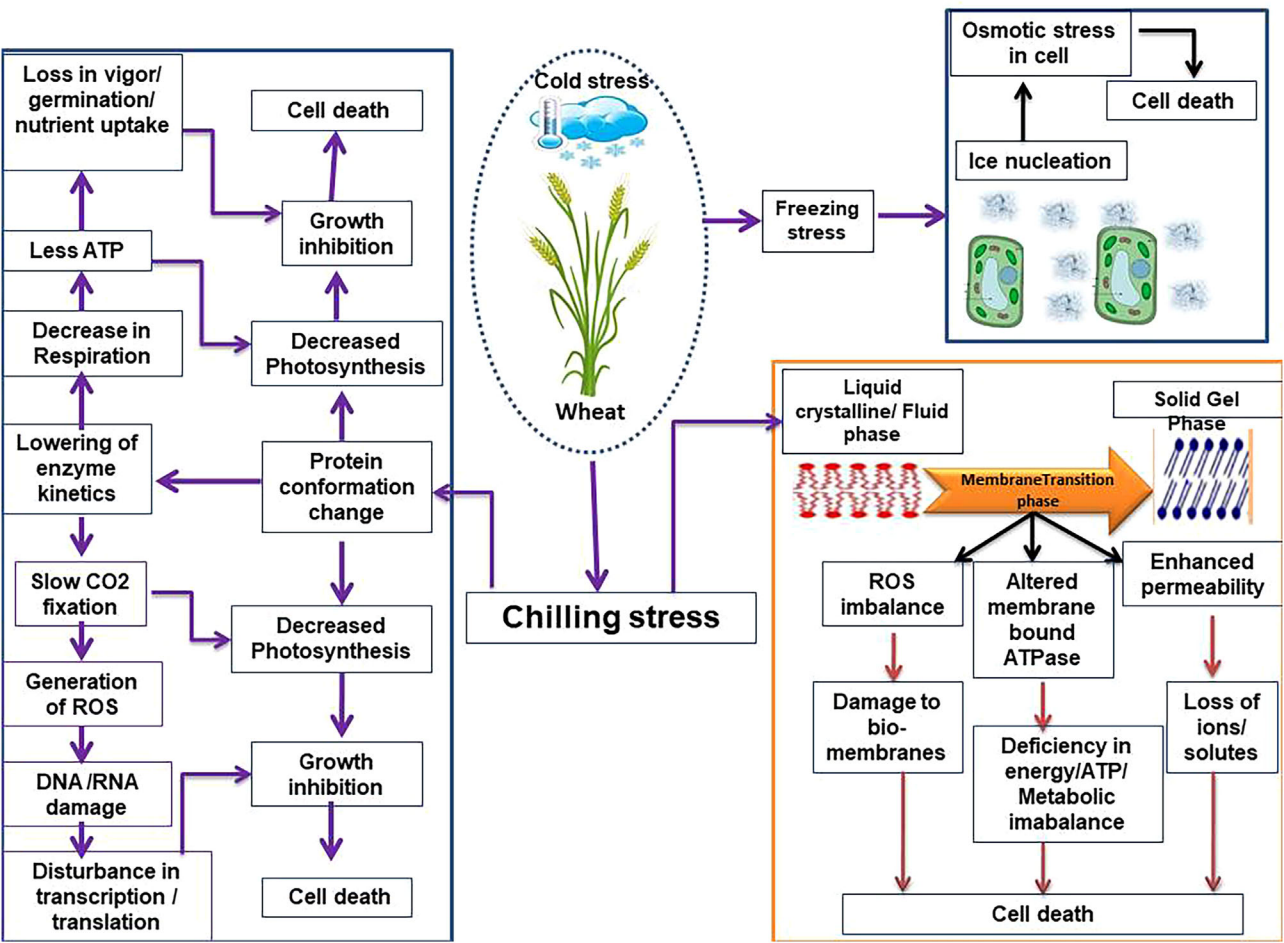
Flow diagram showing the effect of cold stress on cellular processes of plant that leads to cell death ultimately. ROS, reactive oxygen species.

Cold stress is a common abiotic stress that plants experience when exposed to low temperatures. Chilling stress is a type of cold stress that occurs when plants are exposed to temperatures above freezing but below their normal growth temperature range, typically around 0–15°C. However, plants when exposed to temperatures below freezing (0°C), suffer from freezing stress, which is more detrimental to plants than chilling stress (Bracale et al., 2003). During freezing stress, intracellular or extracellular ice crystal formation occurs, which affects the protoplasmic framework of the cell, and when these ice crystals grow large enough, they kill the cell (Lukatkin et al., 2012) (Figure 1). Cold stress leads to cell/ plant death due to the generation of various ROS, namely, superoxide radicals, hydrogen peroxide, hydroxyl radicals, and singlet oxygen, in abundance in chloroplasts, mitochondria, and peroxisomes, which cause oxidative damage to proteins and DNA, peroxidation of membrane lipids, enzyme inhibition, or even cell death (Apel & Hirt, 2004). The major adverse effect of cold stress in plants has been seen in terms of plasma membrane damage due to the production of ROS (Kocsy et al., 2011). This damage occurs due to a change in the plasma membrane's semifluid state to a semi‐crystalline state, resulting in the cessation of protoplast flow and an increase in membrane permeability (Figure 1). This can lead to electrolyte leakage and loss of balance of intracellular ions. The biomembrane system, including the cell membrane, nuclear membrane, and organelle membrane is the initial site of injury, particularly in terms of its structure, function, stability, and enzyme activity, resulting in substantial metabolic imbalance, especially involving respiration and photosynthesis, which ultimately leads to decreased crop yields (Ritonga et al., 2020). So, cold stress induces major changes in the plant cells to which sensitive plants are not able to cope, resulting in their death. However, most plants tend to survive and continue their life cycle under cold stress by developing cold tolerance by exhibiting changes in gene expression; such behavior is termed as cold acclimation (Hassan et al., 2021). It is stated that the counter action of plants to cold stress is carried out through detection (sensing) of stress followed by signal perception, transduction, and induction of cold‐tolerant gene expression, which subsequently initiate the cascade of transcriptional, biochemical, and physiological events vital for cold tolerance in the plant (Heidarvand & Maali Amiri, 2010).

**Core Ideas**

- Cold stress significantly affects plant growth and development, particularly in staple food crops (rice, wheat, and maize) that are often grown in regions with cold climates.
- Understanding the physiological, biochemical, and molecular mechanisms that plants use to respond and adapt to cold stress is crucial for developing cold‐tolerant varieties of staple food crops.
- Advances in various technologies, such as transcriptomics, proteomics, and gene editing, have provided insights into the genetic and molecular mechanisms underlying cold tolerance in staple food crops.
- The knowledge gained from these technological advancements can be applied to develop new breeding strategies and crop improvement techniques to produce cold‐tolerant varieties of staple food crops ensuring food security in regions that experience cold stress.

## 3 COLD SIGNAL PERCEPTION AND TRANSDUCTION IN PLANTS

**FIGURE 2:**
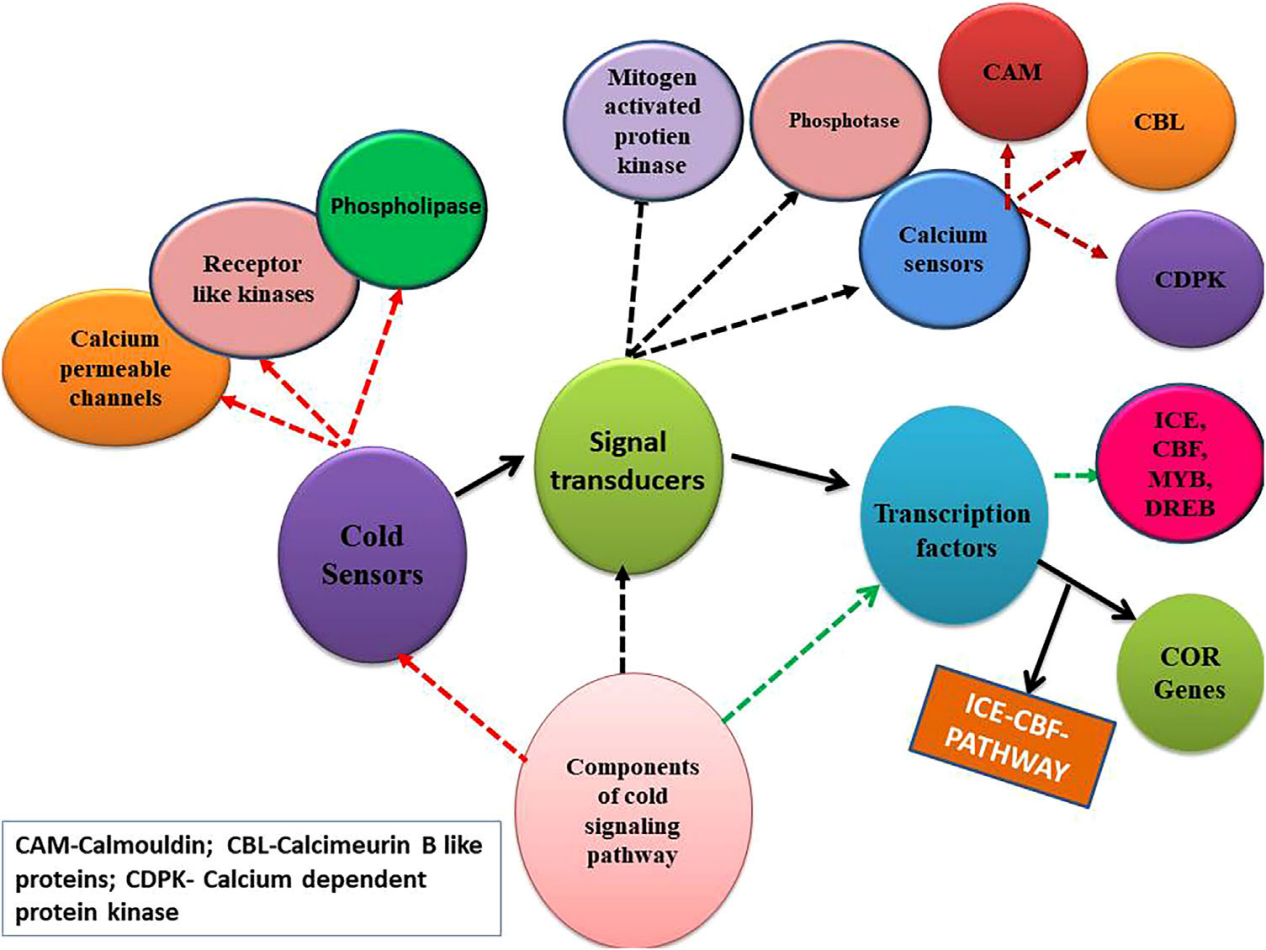
Schematic model showing signaling pathway involved in acquisition of cold tolerance in plants. CBF, C‐repeat/DRE‐binding factor; COR, cold‐responsive; ICE, inducer of CBF expression.

Uncovering the mechanism through which plants perceive and transduce cold signals is crucial for understanding how plants avoid/minimize injury caused by chilling temperatures. In plants, the plasma membrane serves as the central hub for perceiving cold stress, playing a pivotal role in maintaining membrane fluidity and integrity when exposed to low temperatures (Orvar et al., 2000; Solanke et al., 2008; Yadav, 2010). It acts as the frontline sensor, detecting changes in temperature and orchestrating adjustments to ensure the plant's adaptability and survival in challenging cold conditions. This intricate process involves a network of molecular responses and signaling pathways that are finely tuned to safeguard the plant's overall health and functionality. Cold stress reduces membrane fluidity, which affects membrane‐associated cellular functions, thereby making the plasma membrane (PM) the primary sensor of low‐temperature stress (Knight et al., 2012). Microdomains with lipid raft formation and composition, such as sphingolipids in the PM, are utilized by plants to detect specific temperature ranges. Calcium channels, as well as receptor‐like protein kinases, such as two‐component histidine kinases, RLKs, and G‐protein‐associated kinases, play crucial roles in the perception of cold stress. However, calcium channels are a major class of cold stress sensors that allow calcium to enter the cell (Chen et al., 2021). Through the membrane rigidification‐activated mechanosensitive or ligand‐activated Ca2+ channels, cold stress induces a transient Ca2+ influx into the cytosol. Following cold stress perceptions, the cytosol and nucleus of plant cells undergo cold stress signal transduction. Ca2+ and ROS are examples of second messengers that transmit extrinsic cold signals to intracellular signaling systems. The major components underlying cold signal‐sensing/transduction in cereals include calcium signaling, ROS, protein kinase (the mitogen‐activated protein kinase [MAPK] signaling), plant hormone signaling, and lipid signaling cascade (Yadav, 2010) (Figure 2). Here, we discuss the mechanisms underlying some of these key components involved in cold signal sensing and transduction in further detail.

### 3.1 Calcium channels

Calcium (Ca^2+^) is a universal secondary messenger that responds to cold stimuli. The fluctuation in cytosolic Ca^2+^ levels is sensed by calcium‐dependent protein kinases (CDPKs), which modify the phosphorylation status of substrate proteins and mediate the cold stress signaling pathway (Asano et al., 2012; Knight et al., 2001; Ray et al., 2007; Solanke et al., 2008; Thoday et al., 2015; Wilkinis et al., 2016; Zhu et al., 2016). The CDPKs produced under cold stress target significant cold stress‐responsive genes, leading to the adaptation of cereals to unfavorably low temperatures. The CDPK genes that confer stress tolerance in cereals have been identified and are listed in (Table 1). Occasionally, CDPKs confer simultaneous multiple stress tolerance. For instance, overexpression of the rice *OsCDPK7* gene conferred cold, salt, and drought tolerance in transgenic rice plants (Komatsu et al., 2007; Saijo et al., 2000, 2001). In addition to CDPKs, other calcium sensors like calmodulins, calmodulin‐like proteins (CMLs), and calcineurin B‐like (CBL) proteins also act as calcium sensors that capture transient calcium signals in the cytoplasm and transfer these signals to downstream components, causing physio‐biochemical changes necessary for cold stress tolerance (Albrecht et al., 2003).

**TABLE 1:** List of some key sensors and their corresponding genes that regulate signal transduction during cold acclimation in rice, wheat, and maize.

Senor family	Crop	Gene	Reference
Calcium‐dependent protein kinase (CDPK)	Rice	*OsCDPK7*	Saijo et al. (2000, 2001
	Rice	*OsCPK4*	Ray et al. (2007)
	Rice	*OsCPK17*	Almadanim et al. (2017)
	Rice	*OsCPK24*	Liu, Xu et al. (2018)
	Rice	*OsCPk13*	Ray et al. (2007)
	Maize	*Zm*cpk5	Trzcinska‐Danielewicz et al. (2009)
	Maize	*Zm*cpk1	Weckwerth et al. (2015)
	Maize	*ZmCK1*	Wang et al. (2013)
	Wheat	*TaSnRK2.8*	Zhang et al. (2010)
Calcineurin B‐like (CBL) protein	Wheat	*TaCBL*	Sun et al. (2015)
CBL‐interacting protein kinases (CIPK)	Wheat	*Ta CIPK*	Sun et al. (2015)
CIPK	Rice	*OsCIPK3*	Xang et al. (007)
Mitogen activated protein kinase (MAPK)	Wheat	*TaMPK3*	Goyal et al. (2018)
MAPK	Wheat	*TaMPK6*	Goyal et al. (2018)
MAPK	Wheat	*TaMPK4*	Goyal et al. (2018)
MAPK	Rice	*OsMPK3*	Zhang et al. (2016)
MAPK	Maize	*ZmMPK17*	Pan et al. (2015)
MAPK	Maize	*ZmMKK4*	Kong et al. (2011)
MAPK	Maize	*ZmMPK5*	Mira et al. (2021)
Phospholipase C	Wheat	*TaPLC1, TaPLC2*	Khalil et al. (2011)
Inositol polyphosphate 5‐phosphatases (5PTases)		*Os5PTase3a, Os5PTase3b, Os5PTase10b, Os5PTase11, Os5PTase12, Os5PTase13*, and *Os5PTase15*	Faraji et al. (2020)

### 3.2 MAPK signaling

MAPKs regulate cell division, development, metabolism, and stress responses, including cold stress in plants (Moustafa et al., 2014). The proteins of different MAPK subfamilies (MAPKs, MAPK kinases [MKKs], and MAPK kinase kinases [MKKKs]) interact sequentially in the signaling cascade (Goyal et al., 2018). Precisely, MKKKs get phosphorylated in response to environmental or developmental signals and subsequently activate MKKs, which in turn activate MAPKs (or MPKs). MAPKs have been examined as signaling molecules in cold stress adaptation in rice, wheat, and maize (for details, see Table 1).

### 3.3 Phytohormones

Plant hormones like auxin, abscisic acid (ABA), ethylene (ETH), cytokinins (CKs), gibberellins (GAs), jasmonic acid (JA), and brassinosteroids (BRs) modulate cold tolerance via CBF (C‐repeat/DRE‐binding factor)‐dependent and CBF‐independent pathways. In the ABA‐independent pathway/CBF‐dependent pathway, low temperature boosts CBF transcription factors, which activate downstream cold‐responsive genes that increase freezing tolerance in rice, wheat, and maize (Lv et al., 2018; Sun et al., 2009; Verma et al., 2019; Xu et al., 2008). On the other hand, in the ABA‐dependent pathway, endogenous ABA activates basic leucine zipper transcription factors (Uno et al., 2000; Xiong et al., 2002), WRKY transcription factors (Ramamoorthy et al., 2008; Tao et al., 2011), and dehydration responsive element‐binding factors (DBFs) (Xu et al., 2008), and then regulate ABA‐dependent cold‐responsive genes that modulate cold stress in cereals. In addition to ABA, ethylene, jasmonates (JA), and salicylic acid (SA) also induce cold stress tolerance (Zhao et al., 2019) by rapidly raising endogenous JA levels via stimulation of JA biosynthesis genes. This enables ICE1 and ICE2 to activate CBFs by binding to the DRE/CRT box promoter sequence element found in the ICE regulon promoters (Kazan, 2015; Wang et al., 2020).

### 3.4 Lipid molecules

The lipid bilayer membrane separates the cell contents from the outside environment, making them vital in cold signal transmission (Hou et al., 2016). The plasma membrane senses environmental stimuli like cold stress and generates signaling lipids via enzymes, including phospholipases, lipid kinases, and phosphatases. Signaling lipids such as phosphatidic acid (PA), phosphoinositides (PIs), sphingolipids, lysophospholipids, oxylipins, N‐acylethanolamines, and free fatty acids (FFAs) make up less than 1% of total lipids, and their concentrations increase with biotic and abiotic stresses, including cold stress in rice, wheat, and maize (Bargmann et al., 2009; Meijer & Munnik, 2003; Munnik & Vermeer, 2010; Testerink & Munnik, 2005). These signaling lipids cause lipid‐dependent cascade reactions in cold‐exposed plants, activating downstream genes (Table 1) that stimulate adaptation to cold stress (Hou et al., 2016). These genes encode proteins involved in osmolyte biosynthesis, regulation of ion channels, mediation of receptors, participation in calcium signaling components, and the facilitation of various signaling factors or enzymes, which help the plants tolerate cold stress (Tuteja & Sopory, 2008).

## 4 ADVANCES IN OMICS APPROACHES FOR UNCOVERING COLD TOLERANCE IN MAJOR STAPLE CROPS

**FIGURE 3:**
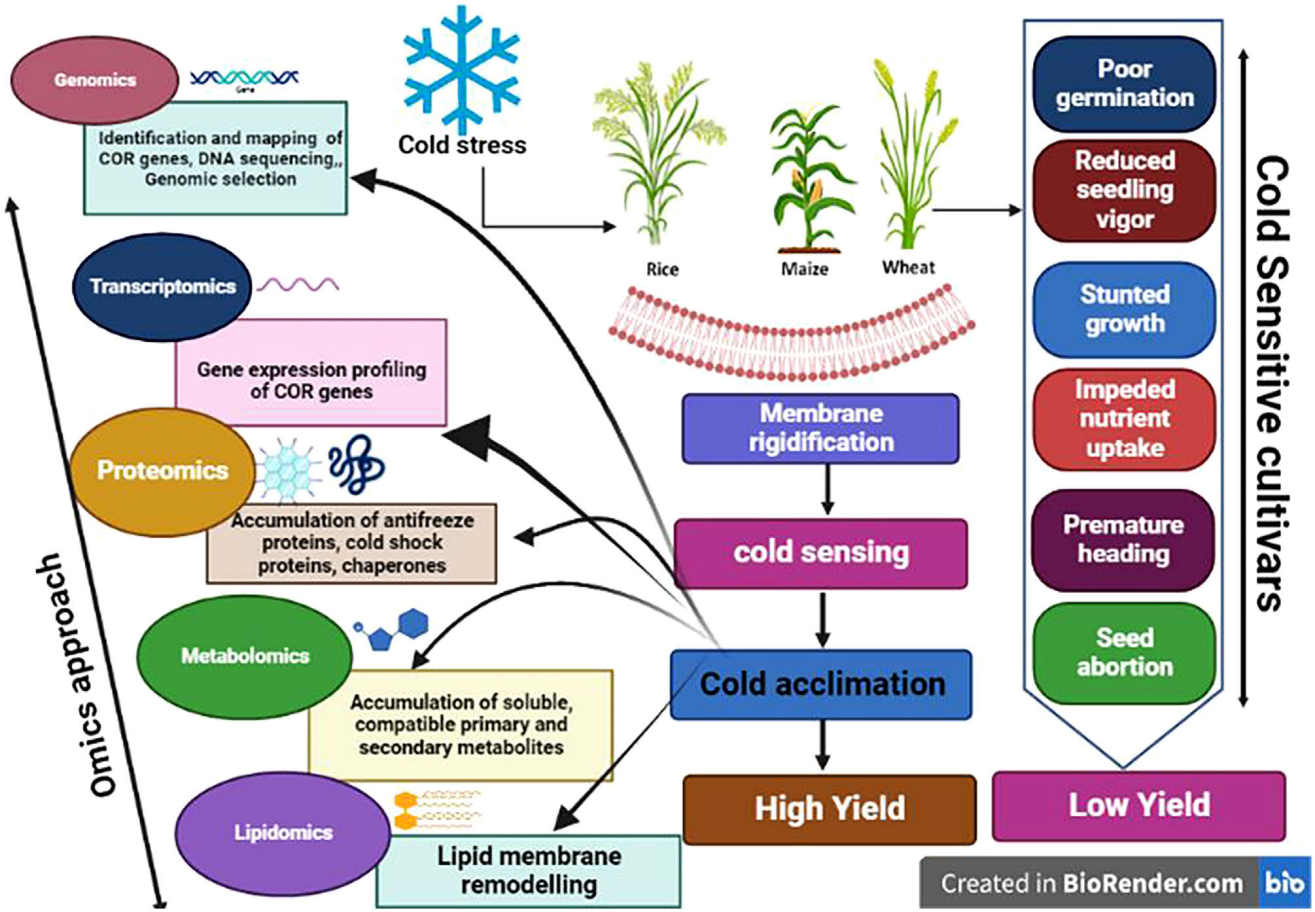
Schematic representation of various omics approaches to study cold acclimation in rice, maize, wheat crops. COR, cold‐responsive.

The last two decades have witnessed an increased use of omics approaches in studying and dissecting complex traits and their integration into breeding programs (Roorkiwal et al., 2020; Varshney, Bohra et al., 2021). The methods to deploy these procedures into plant breeding have improved at lightning speed, and their costs have plummeted, which has made their deployment easy. Cold tolerance particularly in cereals represents one of the most important traits in plant breeding, and various omics approaches have been utilized in studying this complex trait. Here, we highlight some key achievements of omics approaches in dissecting and/or improving cold stress tolerance in major staple crops (Figure 3).

### 4.1 Genomics: Identifying QTLs/genes associated with cold tolerance

Cold tolerance is a complex trait controlled by numerous genes and impacted by a variety of environmental factors, including low temperature. A fundamental aspect of developing cold‐tolerant plant varieties is identifying quantitative trait loci (QTLs) that are associated with cold tolerance. During the early growth stages, comprehending the genetic mechanisms that underpin cold tolerance is essential, as it plays a crucial role in regulating seedling establishment, mortality, and subsequent vegetative growth vigor (Jiang et al., 2008; Lou et al., 2007). In cereal crops, researchers have utilized various mapping populations to perform QTL analyses and discover multiple QTLs responsible for providing tolerance to cold stress during different developmental stages (Table 2). In rice, QTL analysis for cold tolerance at the seedling stage has led to the identification of five QTLs linked to the low‐temperature response, one each on chromosomes 1, 9, and 11, and two on chromosome 3 (Misawa et al., 2000). Similarly, two major QTLs on chromosomes 4 and 12 (Andaya & Mackill, 2003); three QTLs on chromosomes 3, 7, and 11 (Zhang et al., 2005); three QTLs on chromosome 1, 5, and 6 (Jiang et al., 2008); four QTLs on chromosomes 1, 7, and 11 (Cheng et al., 2012); four QTLs on chromosomes 2 and 10 (Raharinivo et al., 2016); four QTLs on chromosome 1, 2, 4, and 5 (Park et al., 2013); and two QTLs on chromosome 8 and 4 (Yu et al., 2018) were found to be associated with cold tolerance in rice. Previous results also indicated that QTLs controlling booting‐stage‐cold tolerance in rice were not associated with seedling cold tolerance (Andaya & Mackill, 2003). Six QTLs for cold tolerance were identified on chromosomes 3, 4, and 12 in rice at the booting stage (Zhu et al., 2015). Because of the problems linked with precise phenotyping of cold tolerance component traits, mapping QTLs for cold tolerance during the booting stage is more difficult. A large number of QTLs identified suggests that cold tolerance in rice is quantitatively inherited and controlled by many genes.

**TABLE 2:** List of some major quantitative trait loci (QTLs) identified in rice, wheat, and maize by QTL mapping using different types of mapping populations.

**Crop**	**QTL name**	**Mapping population**	**Population type**	**Reference**
Rice	*qCTF7*	Eikei88223 × Suisei	Back cross	Shinada et al. (2014).
	*qCTF8*			
	*qCTF12*			
	*qSRS1*	Xiushui 09 × IR2061	Back cross	Li‐rui et al. (2012)
	*qSRS7*			
	*qSRS11a*			
	*qSRS11b*			
	*qCT‐3‐1*	Huanghuazhan (HHZ) × (IR64, PSBRC28, PSBRC66, IR50, OM1723, CDR22)	Multiple interconnected breeding (IB)	Zhu et al. (2015)
	*qCT‐3‐2*			
	*qCT‐3‐3*			
	*qCT‐4*			
	*qCT‐12‐2*			
	*qCT‐4*	Chiyohonami × Hitomebore	Back cross	Endo et al. (2016)
	*qSdGwth14‐10‐1*	Chomrongdhan × Vary botry, Soameva	Back cross (BC1F2)	Raharinivo et al. (2016)
	*qSdGwth14‐10‐2*			
	*qLfGwth14‐10‐1*			
	*qSdVig0‐2‐1*			
	*qSCT8*	Xieqingzao B (*O*. *sativa* L.)× Dongxiang (*O. rufipogon* Griff.)	Backcross inbred line (BIL)	Yu et al. (2018)
	*qSCT4.3*			
	*qSCT4.1*			
	*qSCT4.2*			
	*qSCT12*			
	*qCT4.6*	Chaoyoul× (X22, Yuanjing7, Fengaizhan, Chhomrong, Doddi)	Back cross	Liang et al. (2018)
	*qCT6.6*			
	*qCT11.5*			
	*qCT1.2*			
	*qCT2.4*			
	*qCT3.5*			
	*qCT3.12*			
	*qCT4.2*			
	*qCT4.6*			
	*qCT6.6*			
	*qCT9.7*			
	*qCT11.5*			
	*Qct1*	Kirara397 × Hatsushizuku	Recombinant inbred line (RIL)	Kuroki et al. (2009)
	*Qcl2*			
	*Qct10*			
	*Qctb1‐1*	Kunmingxiaobaigu (KMXBG) ×Towada	Near‐isogenic line (NIL)	Zeng et al. (2009)
	*Qctb4‐2,4‐3,4‐4*			
	*Qctb4‐1,4‐5*			
	*Qctb5‐1*			
	*Ctb1*	KMXBG× Towada	F_2_‐F_3_	Zhou et al. (2010)
	*QTL2.1*	Dasanbyeo×TR22183	RIL	Jiang et al. (2008)
	*QTL8.1*			
	*QTL10.1*			
	*qFERCT2*	Diversity panel	400 accessions	Shakiba et al. (2017)
	*qFERCT6‐1*			
	*qFERCT6‐2*			
	*qFERCT6‐3*			
	*qFERCT6‐4*			
	*qFERCT7*			
	*qFERCT12*			
	*qCTB10 ‐ 2*	KMXBG × Towada	Back cross	Li, Fu et al. (2018)
	*QRCT7*	KMXBG × Towada	F2	Dai et al. (2004)
	*QRCT10*			
	*qCT‐1*	Koshihikari × Akihikari	Doubled haploid lines (DHL)	Takeuchi et al. (2001)
	*Qcl‐1*			
	*QCT‐7*			
	*QCT‐11*			
Wheat	*5A QTL*	Norstar × Manitou	DHL	Baga et al. (2007)
	*QFrbr.wak‐5A*	Brundage × Coda	RIL	Case et al. (2014)
	*QFrbr.wak‐5B*			
	*QFrbr. wak‐3A*			
	*QFrco.wak‐2A*			
	*QFrco. wak‐6D*			
	*QLT50.usw‐1B*	Norstar × Manitou	DHL	Fowler et al. (2016)
	*QLT50.usw‐1D*			
	*QLT50.usw‐2B*			
	*QLT50.usw‐5A.1nm*			
	*QLT50.usw‐5A.2nm*			
	*QLT50.usw‐5B*			
	*QLT50.usw‐3A*	CappelleDesprez × Norstar		
	*QLT50.usw‐5A.1nc*			
	*QLT50.usw‐5A.2nc*			
	*QLT50.usw‐7B*			
	30 QTLs	Bethlehem × Westonia (BW); Gregory × Bethlehem substitution line 7AS (G7A); Spitfire × Bethlehem substitution line 7AS (Sp7A)	DHL	Zhang et al. (2022)
	7 QTLs	Norstar× Zagros	F2:3	Sofalian et al. (2009)
	7 QTLs	Diversity panel	450 Canadian wheat varieties	Chen et al. (2019)
	53 marker‐trait associations (MTAs)	Diversity panel	276 winter wheat genotypes	Soleimani et al. (2021)
	76 MTAs	Diversity panel	543 wheat accessions	Zhao et al. (2020)
Maize	*QTL‐7*	EP42 (flint) × A661 (dent)	F2:3	Rodríguez et al. (2014)
	*QTL‐8*			
	*QTL‐9*			
	*QTL‐10*			
	*QTL‐1*	B73 × Mo17	IBM RIL population syn 4	Goering et al. (2021)
	*QTL‐2*.			
	*qLTGR5‐1*		IBM RIL population syn 4	Hu et al. (2016)
	*qLTPRL9‐1*			
	One QTL	ETH‐DH7 × ETH‐DL3	F2:3	Fracheboud et al. (2004)
	13 QTLs	B73 × Mo17 (IBM) Syn10	DHL	Han et al. (2022)
	20 QTLs	Lo964 × Lo1016	F2:4	Hund et al. (2004)
	*bins3.01*	B73 × Mo17	RIL	Rodriguez et al. (2008)
	*bins6.03*			
	27 QTLs	B73 × P39	RIL	Allam et al. (2016)
		B73 × IL14		
	275 MTAs	Diversity panel	306 dent and 292 European flint maize inbred lines	Revilla et al. (2016)
	187 MTAs	Diversity panel	836 maize inbred lines	Yi et al. (2021)

In wheat, the genetic control of frost tolerance is complex, and at least 10 of the 21 chromosome pairs are involved in the regulatory gene network. Major genes affecting winter hardiness have been mapped on the long arms of homoeologous group 5 chromosomes (Sutka, 2001). The major frost tolerance locus, *Fr‐A1* (formerly *Fr1*), was mapped on the long arm of chromosome 5A, while another locus for frost tolerance designated *Fr‐A2* was mapped on the long arm of chromosome 5A of diploid wheat (*Triticum monococcum*), 40 cM from the centromere and 30 cM proximal to the major frost tolerance locus *Fr‐A1* (Vagujfalvi et al., 2003). The QTL on chromosome 5A is located 46 cM proximal to the vernalization locus *vrn‐A1* (Baga et al., 2007). In addition to this major cold tolerance locus on 5A, several QTLs controlling cold tolerance have been identified on other wheat chromosomes, including 1D, 2B, 4B, 4D, 5D, 6A, 7A, 7B, and 7D (Fowler et al., 2016; Francia et al., 2004; Snape et al., 2001; Sofalian et al., 2009; Taleei et al., 2010). In addition to the QTL for freezing tolerance, QTLs for snow mold tolerance were also found to co‐localize on wheat chromosome 5A, indicating that this QTL has a pleiotropic effect (Kruse et al., 2017). Similarly, a QTL on chromosome 6A of wheat for minimum final leaf number, which determines the rate of phenological development at the seedling stage, was linked to a QTL for low‐temperature tolerance, grain quality, and agronomic characteristics expressed up to the time of maturity (Fowler et al., 2016). At the young microspore developmental stage, 30 major QTLs for frost tolerance were detected on 17 chromosomes, including 2A, 2B, 2D, 3A, 4A, 4B, 4D, 5A, 5D, 6D, 7A, 7B, and 7D in wheat. Many of these QTLs were linked to anthesis and maturity QTLs, as well as anthesis‐related genes, indicating that frost tolerance in wheat occurs around late flowering, when the plant has escaped from the risk of frost events (Zhang et al., 2021). In contrast, the early flowering genes *Vrn‐B1a* and *TaFT3‐1B* on chromosomes 5B and 1B, respectively, did not lead to frost damage since no QTL was identified on the two loci, indicating that these two early‐flowering genes are associated with frost tolerance and can be utilized in breeding (Zhang et al., 2021).

Numerous QTLs controlling cold tolerance‐related traits like germination, leaf area, chlorophyll content, the maximum quantum efficiency of photosystem II (Fv/Fm), CO_2_ fixation, the ratio of fresh aboveground weight/root weight, and root architecture were detected across 10 chromosomes in maize seedlings (Frascaroli & Revilla, 2018; Hu et al., 2016; Revilla et al., 2016; Rodríguez et al., 2014; Strigens et al., 2013; Yan et al., 2017). Most of these QTLs are located in specific genomic regions, particularly bin 10.04 (Yi et al., 2020). In addition, three genomic regions on chromosomes 2, 4, and 8 that regulate the early development of maize seedlings under cold conditions have also been identified (Rodríguez et al., 2014).

To sum up, several QTLs identified using the conventional QTL mapping approach suggested that cold tolerance in cereals is quantitatively inherited and controlled by many genes. Still, we believe these identified QTLs are an underestimation because the conventional QTL mapping approaches generally involve bi‐parental mapping populations and are limited by low resolution. However, the exploitation of single nucleotide polymorphisms (SNPs) and genotyping‐by‐sequencing (GBS) has made it possible to search for more QTLs controlling cold tolerance in cereals through genome‐wide association studies (GWASs). In wheat, GWAS analyses led to the identification of seven QTLs associated with cold tolerance using a diversity panel of 450 Canadian wheat varieties (Chen et al., 2019). Also, 23 QTLs localized on 11 chromosomes (1A, 1B, 2A, 2B, 2D, 3A, 3D, 4A, 5A, 5B, and 7D) were identified by GWAS using a diverse panel of 276 winter wheat accessions (Soleimani et al., 2022). Using GWAS, one major additive effect QTL was identified on wheat chromosome 5B (Zhao et al., 2013). Similarly, numerous QTLs associated with chilling tolerance have been identified in rice with GWAS (Fujino et al., 2015; Ham et al., 2021; Jeong et al., 2021; Li, Liu et al., 2021; Lv et al., 2016; Pan et al., 2015; Shakiba et al., 2017; Wang, Liu et al., 2016; Zhang et al., 2022). In maize, many QTLs associated with cold tolerance were identified using the GWAS approach. However, these QTLs explained low amounts of phenotypic variance for early growth and chlorophyll fluorescence (Revilla et al., 2016; Strigens et al., 2013). Very recently, a large association panel of 836 inbred maize lines revealed a broader genetic diversity for cold tolerance. Predominantly, favorable QTLs with small effects were identified, indicating that genomic selection is the most promising option for breeding maize for cold tolerance (Yi et al., 2021). As the underlying trait is complex in nature and regulated by many genes, these findings for cold tolerance will open new avenues for the genetic improvement of rice, wheat, and maize genotypes through marker‐assisted or genome‐wide selection.

### 4.2 Genetic regulation of cold acclimation in cereals

The cold acclimation process involves differential regulation of a variety of genes. For instance, many cold‐responsive genes have been found in plants and are classified as dehydrin (DHN), late embryogenesis abundance (LEA), cold‐responsive (COR), and responsive to abscisic acid (RBA) (Guo et al., 2019). Transcriptional activators, which activate cold tolerance structural genes, are triggered by low temperatures. In *Arabidopsis*, the transcription factor *ICE1* (Inducer of CBF Expression), which encodes MYC‐like basic helix‐loop‐helix (bHLH) protein, is activated by a low‐temperature signaling pathway that subsequently activates the CBF (C‐repeat‐Binding Factor) protein family (Chinnusamy et al., 2010). In addition to *ICE*, several other genes and proteins affect *CBF* expression. The expression of CBF is up‐regulated by the *LOS4* gene, which encodes a DEAD‐box RNA helicase (Gong et al., 2002). In contrast, other genes such as *FRY2* (Xiong et al., 2002) and *HOS1* (Lee et al., 2001) down‐regulate *CBF* expression.

Three transcriptional activators, *CBF1*, *CBF2*, and *CBF3*, have been reported to activate cold tolerance genes (Gilmour et al., 2000). To this end, constitutive expression of *CBF2* suppresses *CBF1* and *CBF3* expression (Novillo et al., 2004), and increased expression of *CBF1* and *CBF3* occurs early during cold acclimation. The high amount of *CBF1* and *CBF3* expression represses *CBF2* (Novillo et al., 2004), which in turn represses *CBF1*, followed by *CBF3* activating the transcription of COR genes. Over time, the levels of *CBF3* and *CBF1* genes decrease, allowing *CBF2* to express. The CBF protein family is present in many plants, including *Arabidopsis* (Chinnusamy et al., 2010), barley (Skinner et al., 2005), and wheat (Jaglo et al., 2001). Miller et al. (2006) recently reported that there are 13 different *CBF* genes in wheat, 11 of which are present in the same region of chromosome 5 containing the *Fr‐A2* locus. Furthermore, *Wcs120, WCS19, Wcor15*, *WCOR39*, and *Wcor410* represent some examples of the COR genes in wheat (Repkina et al., 2021; Talanova et al., 2018; Vítámvás et al., 2021), which are activated by the CBF protein family. The induction of COR genes by low‐temperature stress is regulated by CBF and ABA‐responsive element binding protein (AREB) (Zhou et al., 2010). Pre‐treatment of seedlings with salicylic acid enhanced wheat freezing tolerance by up‐regulating the expression of both ABA‐dependent (*ABI5* and *RAB17*) and ABA‐independent COR genes (*CBF3*, *COR14*, *CS120*) (Wang, Wang et al., 2018). Also, after 3 and 11 weeks of cold treatment, *COR14b*, *CS120*, and *Wdhn13* genes tended to be highly expressed in freezing‐tolerant RILs than in freezing‐susceptible RILs, suggesting that they are important for freezing tolerance (Kruse et al., 2020).

Over the past two decades, many studies have revealed that cold stress responses in rice involve complex regulatory networks (Guo et al., 2018; Zhou et al., 2018). A few genes conferring cold tolerance, including *COLD1*, *qLTG3–1*, *LTG1*, *Ctb1*, *CTB4a*, and *LTT7* were identified in rice (Ma et al., 2015). Among these genes, *qLTG3–1* encodes an unknown protein that is tightly associated with the vacuolation of tissues covering the embryo and was shown to improve cold tolerance in rice at the germination stage (Fujino et al., 2008); also, *LTG1* that encodes a casein kinase and plays an important role in the adaptive growth of plants (Gong et al., 2002). The overexpression of *LTG5* (low‐temperature growth 5) gene that encodes a UDP‐glucosyltransferase conferred cold tolerance in *indica* rice (Pan et al., 2020). A point mutation in the *low‐temperature tolerance 1* (*LTT1*) gene was found to improve cold tolerance by maintaining tapetum degradation and pollen development through activating systems that metabolize ROS (Xu et al., 2020). Another major QTL for cold tolerance at the booting stage, CTB4a (Xu et al., 2008), that encodes a conserved leucine‐rich repeat receptor‐like kinase was identified using a map‐based cloning approach (Li et al., 2021; Xiao et al., 2018; Zhang, Zhang et al., 2017, Zhang, Li et al., 2017). Over‐expression of the rice *CBF* homolog, *OsDREB1A*, has shown enhanced tolerance of transgenic rice to drought, high‐salt, and low‐temperature stresses (Ito et al., 2006). In transgenic rice plants, over‐expressing *DaCBF7*, a *CBF* gene from *D. antarctica*, resulted in enhanced cold tolerance compared to wild‐type plants (Byun et al., 2018). Expression of another *D. antarctica CBF4* gene (*DaCBF4*), which encodes a homolog of cereal CBF group IV gene, resulted in a cold‐specific phenotype in rice similar to that of *DaCBF7*‐overexpressing plants (Byun et al., 2018). *OsDREB1G*, a functionally unidentified member of the *DREB1* subgroup, specifically functions in the cold stress response and thus could be useful for developing transgenic rice with enhanced cold‐stress tolerance (Moon et al., 2019). Yet another gene, *OsROC1*, was identified to enhance chilling tolerance by activating *OsDREB1* in rice (Dou et al., 2016).

In the case of maize under chilling conditions, the expression level of the ABA stress‐ripening (ASR) gene, *ZmASR1*, was found to be high in chilling‐tolerant maize cultivars and resulted in enhanced chilling tolerance (Li, Pan et al., 2018). Also, an Argonaute (AGO) protein, named ZmAGO, and the MYB transcription factor gene, *ZmMYB31*, were found to be up‐regulated in response to cold stress in maize (Zhai et al., 2019).

### 4.3 Transcriptomics: Interpreting the functional elements of the genome under low temperatures

The advent of high‐throughput sequencing technologies has revolutionized our understanding of plant responses to cold temperatures. By analyzing the comprehensive transcriptome data obtained through these technologies, researchers can identify candidate genes that are involved in low‐temperature responsiveness, a critical factor in plant adaptation to cold environments. Recent RNA‐seq data analysis of rice, wheat, and maize has revealed several candidate genes that play pivotal roles in cold acclimation and freezing tolerance. This knowledge is particularly valuable for developing crops that are better adapted to cold temperatures, a crucial factor for food security in regions where low temperatures can limit plant growth and productivity. The identification of cold‐related genes in wheat, for instance, has shed light on the molecular mechanisms underlying the plant's response to cold stress, including the role of dehydrin, LEA proteins, carbohydrate and amino acid synthesis proteins, and EF‐hand proteins in regulating the transcription of downstream defensive genes. Genome‐wide transcriptional profiling in the crowns of field‐grown spring and winter wheat genotypes revealed multiple signaling and interactive pathways that influence cold tolerance and phenological development to optimize plant growth and development upon exposure to cold stress (Li, Li et al., 2018). Transcriptome analysis of durum wheat cultivar “CBW 0101” subjected to low temperatures during the reproductive stage revealed differential expression of transcription factors similar to that observed in winter wheat. Further, long noncoding RNAs (lncRNAs) were increased in cold‐stressed plants, suggesting a role for lncRNA molecules in response to low temperatures in durum wheat (Diaz et al., 2019).

Transcriptome research in cold‐tolerant and susceptible rice genotypes revealed that membrane transportability, sucrose, antioxidant production, and Ca^2+^ signaling are enhanced in cold‐tolerant seedlings, while cold stress causes cold‐sensitive seedlings to synthesize heat shock proteins and dehydrins (Dametto et al., 2015; Shen et al., 2014). Also, comprehensive transcriptome profiling of two cold‐tolerant and susceptible rice genotypes, 253 (cold‐sensitive) and Y12–4 (cold‐tolerant), at the germination stage revealed that Y12–4 up‐regulated calmodulin family protein, cytochrome P450 family protein, ethylene response factor, jasmonate zinc‐domain protein, NB‐ARC domain‐containing protein, serine/threonine protein kinase, cold shock protein, MYB transcription factor, UDP‐glucosyltransferase signal transduction, and energy metabolism genes (Pan et al., 2020). Under cold stress, RNA‐seq detected many common and distinct DEGs in cold‐tolerant and cold‐sensitive rice genotypes. Some genes associated with cold stress, such as bHLH and LRR, were found to be enriched in cold‐tolerant genotypes than in cold‐sensitive genotypes (Guan et al., 2019).

Similar to rice and wheat, maize seedlings at low temperatures showed differential regulation of genes involved in photosynthesis, MAPK signaling pathway, plant hormone signal transduction, circadian rhythm, secondary metabolite‐related pathways, ribosome, proteasome, and phenylpropanoid biosynthesis pathway (Yu et al., 2021). In the chilling‐tolerant maize cultivars, genes associated with secondary metabolism and unsaturated fatty acid production were amplified, thereby protecting photosystems and maintaining cell membrane stability (Li et al., 2019; Sobkowiak et al., 2014). RNA‐seq of 276 double haploid lines derived from the European flint landrace “Petkuser Ferdinand Rot,” which differed for cold tolerance showed that ROS detoxification is associated with cold tolerance, while amino acids have played a crucial role as antioxidant precursors and signaling molecules (Frey et al., 2020). To study the interplay between cold stress and a rhythmic cue, the transcriptomic response of maize seedlings to low temperatures in the context of diurnal gene expression was investigated. It was found that severe cold deregulates the circadian gene expression pattern, modulating the transcription of genes related to photosynthesis and other essential biological processes (Jonczyk et al., 2017).

### 4.4 Proteomics: A key to understand protein turnover in response to cold stress

To understand how plants respond to cold stress, it is important to examine the turnover of proteins under such conditions. Cold stress induces the accumulation of various proteins in both chilling tolerant and sensitive plants, as has been demonstrated in several studies (Chen et al., 2015; Grimaud et al., 2013; Koehler et al., 2012; Xu et al., 2013). By analyzing the changes in protein expression and turnover under cold stress conditions, researchers can identify key regulatory pathways and candidate genes that can be targeted to improve the cold tolerance of crops. Proteomics is a valuable tool that can be used to identify stress‐inducible proteins and gain insights into the physiological and stress response mechanisms in plants (Zhang et al., 2017b).

Protein profiling can be performed using gel‐based techniques such as 2D‐gel electrophoresis, 2D differential gel electrophoresis, and gel‐free approaches like isotope‐coded affinity tags, isobaric tags for relative and absolute quantitation (iTRAQ), and stable isotope labeling by amino acids in cell culture (Yates et al., 2009). Numerous proteomic studies have been published in recent years dealing with cereals exposed to cold stress. For instance, proteomics analysis of an Iranian spring wheat cultivar “Kohdasht” exposed to low temperatures (4°C) showed an increase in ascorbate recycling (dehydroroascorbatereductase and ascorbate peroxidase), protein processing (proteasome subunit and cysteine protienase), and tetrapyrrole synthesis (glutamate semialdehydeaminomutase) proteins (Rinalducci et al., 2011). Some Krebs cycle enzymes (isocitrate dehydrogenase, malate dehydrogenase) and photosynthesis‐related proteins (oxygen‐evolving complex proteins, ATP synthase subunits, ferredoxin NADPHoxidoreductase, and some Calvin cycle enzymes) were found to be down‐regulated during cold stress (Rinalducci et al., 2011). Analysis of MALDI‐TOF iTRAQ‐based protein profiling coupled with non‐redundant protein database searches allowed the identification of numerous cold‐induced proteins in wheat, such as pathogenesis‐related proteins, cold‐regulated proteins, cold‐responsive LEA/RAB‐related COR proteins, oxygen‐evolving enhancer proteins, oxalate oxidase, glycolysis enzymes, redox metabolism (thioredoxin‐dependent peroxidase), chaperones, and defense‐related proteins (protein similar to thaumatin) (Gharechahi et al., 2014; Kosova et al., 2013; Zhang et al., 2017). Similarly, iTRAQ‐based quantitative proteomic analysis of maize seedlings under cold stress identified proteins involved in posttranslational modifications, signal transduction, lipid metabolism, inorganic ion transport and metabolism, and other biological processes (Wang, Shan et al., 2016).

Two‐dimensional gel electrophoresis (2‐DE) and isobaric tag labeling were employed to monitor the proteome response of rice to cold stress. Here, proteins involved in energy metabolism, transport, photosynthesis, precursor metabolite production, histones, and vitamin B biosynthesis were found to be differentially regulated under cold stress (Neilson et al., 2011). In cold‐tolerant rice genotypes, proteins involved in ATP synthesis, photosystems, reactive oxygen species production, stress responses, and cell growth and integrity responded quickly to cold stress, preventing cell death (Wang, Wang, Huang, et al., 2018). Label‐free quantitative proteomics in Dongxiang wild rice (*Oryza rufipogon* Griff) and cold‐sensitive cultivated rice “Xieqingzao B” (*Oryza sativa* L. ssp. *indica*) showed the presence of 101 and 216 differentially expressed proteins (DEPs) in cold‐tolerant and cold‐sensitive genotypes, respectively, after cold stress (Liu et al., 2021). Using proteome analysis, researchers investigated various molecular adaptation mechanisms to cold stress in rice. For instance, 60 proteins were found to be enriched in response to decreasing temperatures in rice. MALDI‐TOF/MS discovered cold‐responsive enzymes, cell wall biosynthesis enzymes, energy pathway proteins, and a signal transduction protein (Cui et al., 2005). Novel proteins, including acetyltransferase, phosphphogluconate dehydrogenase, NADP‐specific isocitrate dehydrogenase*, fructokinase*, PrMC3, putative alpha‐soluble NSF attachment protein, and glyoxalase 1, were found to be involved in energy production and metabolism, vesicular trafficking, and detoxification in rice root tissues under chilling stress (Lee et al., 2009).

### 4.5 Metabolomics: Defining characteristic metabolite fingerprints underpinning cold tolerance

Metabolomics represents a powerful approach for detecting and analyzing differentially expressed metabolites in plants in response to cold stress, which can shed new light on the plant's responses to cold stress (Clemente‐Moreno et al., 2020). To date, a large number of metabolites have been identified that can contribute to cold stress tolerance. These metabolites generally function as osmolytes, such as proline, betaine, raffinose, sugars, and amino acids (Zhao et al., 2019); compatible solutes (e.g., sugars and amino acids); or chelating agents. Such metabolites facilitate the tailoring of membrane lipid composition to optimize its liquid/crystalline physical structure necessary for proper functioning under cold stress (Khare et al., 2020; Singh et al., 2020; Yang et al., 2020). In the cold acclimation process, active reconfiguration of the metabolome seems to be achieved in part by changes in cold‐regulated gene expression initiated by signaling cascade, such as the CBF cold response pathway in *Arabidopsis* (Cook et al., 2004; Guy et al., 2008), changes in transcript abundance, and regulatory processes independent of transcript abundance (Kaplan et al., 2007). Comparative metabolomic studies in major staple crops such as wheat, rice, and maize have shown quantitative and qualitative differences in primary metabolites involved in osmoprotection after exposure to cold stress (Pradhan et al., 2019; Sun et al., 2016; Yang et al., 2019; Zhao et al., 2021).

## 5 PRACTICAL IMPLICATIONS AND APPLICATIONS

While our review primarily focuses on elucidating the molecular mechanisms underlying plant responses to cold stress, it is essential to underscore the profound significance of this knowledge in the context of enhancing cold tolerance in staple crops. Understanding these mechanisms not only enriches our scientific understanding but also offers promising avenues for practical applications with the potential to revolutionize agriculture in the face of challenging cold environments.

### 5.1 Precision breeding for cold tolerance

The insights gleaned from the intricate interplay of genes, pathways, and regulatory elements in response to cold stress provide invaluable guidance for precision breeding programs. By identifying and selecting specific genes associated with enhanced cold tolerance, we can expedite the development of crop varieties capable of thriving in colder climates. This knowledge enables breeders to make informed decisions in selecting parental lines with the desired genetic traits for cold tolerance.

### 5.2 Targeted genome editing

Recent advances in genome editing techniques, such as CRISPR‐Cas9, offer the potential to precisely modify key genes linked to cold tolerance. Armed with a deeper understanding of the genetic markers and regulatory elements governing cold stress responses, scientists and breeders can employ genome editing to engineer crops with enhanced resilience to cold temperatures.

### 5.3 Innovative crop management

The molecular insights into cold stress responses inform innovative crop management strategies. These include optimizing planting schedules, fine‐tuning agronomic practices, and implementing stress mitigation measures based on a more profound comprehension of how cold stress impacts various aspects of plant growth and development.

### 5.4 Biotechnological solutions

The identification of crucial regulatory proteins, enzymes, or metabolites involved in cold stress responses opens the door to biotechnological interventions. These may involve the application of specialized compounds or the engineering of beneficial microorganisms that promote cold tolerance in crops.

### 5.5 Climate‐resilient agriculture

As we confront the challenges of climate change, understanding how plants perceive and respond to cold stress is pivotal for adapting agriculture to shifting climate patterns. This knowledge informs the selection of appropriate crop varieties and the development of climate‐resilient agricultural systems that can thrive in increasingly unpredictable weather conditions.

By integrating these practical considerations into our exploration of the molecular intricacies of cold stress responses, we bridge the gap between fundamental research and real‐world applications. This synergy holds the potential to not only bolster our scientific understanding but also transform the agricultural landscape by equipping us to address the pressing issue of cold stress in staple crop production.

## 6 CONCLUSIONS AND FUTURE PERSPECTIVES

Cold stress represents one of the most damaging abiotic stresses with a complex feature, which causes changes in several plant processes at the genome, transcriptome, proteome, and metabolome levels. Being stage‐specific, cold tolerance might appear to be a mechanism regulating cell stability in response to stimuli. Although most crops have the ability to withstand some variations in temperatures, prolonged exposure to cold conditions can cause severe damage to these crops. To minimize such damages, crops undergo a range of biochemical and molecular changes in response to cold stress. The cold signaling pathway in primary staple crops has been explored to some extent over the last few years; however, the molecular mechanisms underlying cold signal sensing and transduction remained largely unclear. Therefore, future research is needed to determine the downstream regulators in the cold signaling pathway, mainly protein kinases and phosphatases.

Even though the global average temperature continues to soar, cold extremes and climatic fluctuations are also increasing throughout the world. Provided the scope of environmental conditions where agriculture is practiced, crops can encounter very discrete cold scenarios, varying from mild to severe cold stress. To expand the area under cultivation of major staple crops to high altitudes and latitudes, there is a rising demand to develop improved varieties that can tolerate cold stress. To meet these pressing requirements, an upcoming challenge will be to develop state‐of‐the‐art omics technologies and deploy them to precisely identify genetic/molecular factors associated with desired cold tolerance component traits and breeding systems. Implementation of genomics has already advanced the breeding process, which is now poised to undergo a quantum leap in the accuracy of choosing parents, determining haplotypes, scrutinizing progeny for a preferred combination of haplotypes, and estimating performance based on genetic evidence (Varshney, Bohra et al., 2021). As a result, integrating recent innovations in omics approaches with conventional breeding efforts will play an important role in developing cold‐tolerant crops for the future. For instance, identifying QTLs for cold tolerance using high‐density mapping populations and GWAS and deploying them in elite varieties using genomics‐assisted breeding can facilitate the development of improved cereal varieties (Varshney, Bohra, Yu et al., 2021). In addition, genome editing of earlier‐identified cold‐stress regulators will serve as an important strategy for increasing cold tolerance in major staple crops. However, non‐politicized regulatory guidelines for genome editing will be critical for scaling such scientific developments to farmers’ fields within a limited time frame. The knowledge of the genes involved in conferring cold tolerance will not only feed the human needs on earth but will also help with the human ambitions to inhabit space, which is part of a long‐term survival plan. Also, the plants with the capabilities to acclimatize quickly under cold stress or withstand the chilling stress might perform better after long‐term cryopreservation, which again would be required to conserve seeds of desirable plant types for the long‐term survival of the human race on this planet.

## AUTHOR CONTRIBUTIONS

**Sofora Jan**: Conceptualization; writing—original draft. **Sachin Rustgi**: Investigation; supervision; writing—review and editing. **Rutwik Barmukh**: Writing—review and editing. **Asif B. Shikari**: Writing—review and editing. **Brenton Leske**: Writing—review and editing. **Amanuel Bekuma**: Writing—original draft. **Darshan Sharma**: Writing—review and editing. **Wujun Ma**: Writing—review and editing. **Upendra Kumar**: Writing—review and editing. **Uttam Kumar**: Writing—review and editing. **Abhishek Bohra**: Writing—review and editing. **Rajeev K. Varshney**: Conceptualization; supervision; Writing—review and editing. **Reyazul Rouf Mir**: Conceptualization; supervision; writing—original draft.

## CONFLICT OF INTEREST STATEMENT

The authors declare no conflict of interest.

## Data Availability

All the data are included in this manuscript.
